# National Hospital for the Paralysed and Epileptic

**Published:** 1902-07-12

**Authors:** 


### NATIONAL HOSPITAL FOR THE PARALYSED AND EPILEPTIC.

THE YEAR'S WORK.

The number of in-patients treated during 1901 was 952, the average number of beds occupied was 177, and the cost per bed was £84 2s. 5d. There were 6,150 out-patients, and the total number of attendances was 37,971. The average cost of each out-patient was 5s. 2½d.

The receipts were: Ordinary, £11,450 8s. 1d.; extraordinary, £11,631 15s. 9d., of which £2,231 5s. 9d. consisted of legacies. Patients' payments amounted to £2,801 16s. 3d.; £2,079 8s. 6d. of this came from the contributing wards, and £722 7s. 9d. from out-patients. Annual subscriptions were £1,537 7s.; donations, £1,325 19s. 5d. The total amount received from King Edward's Fund during 1901 was £1,800, the contributions being paid for the previous year as well; £100 of this was specially for the country branch. From the Sunday Fund £1,475 (for two years) was received, and from the Saturday Fund £235 18s. The ordinary expenditure was £16,570 19s. 1d.; extraordinary, £6,000 7s. 6d. Of this the largest item was "repayment of loans from Commemoration Fund." The sum of £1,116 17s. 6d. was paid in pensions, from the Pension Fund for the incurable; and £293 6s. 11d. was paid in annuities, from the "Donations carrying Life Annuities Fund."

